# Supplementary figures and images for: Glaucoma-Associated CDR1 Peptide Promotes RGC Survival in Retinal Explants through Molecular Interaction with Acidic Leucine Rich Nuclear Phosphoprotein 32A (ANP32A)
Source: Biomolecules. 2023 Jul 22;13(7):1161. doi: 10.3390/biom13071161 (PMC10377047; doi:10.3390/biom13071161)

**Protein  
phosphorylation**

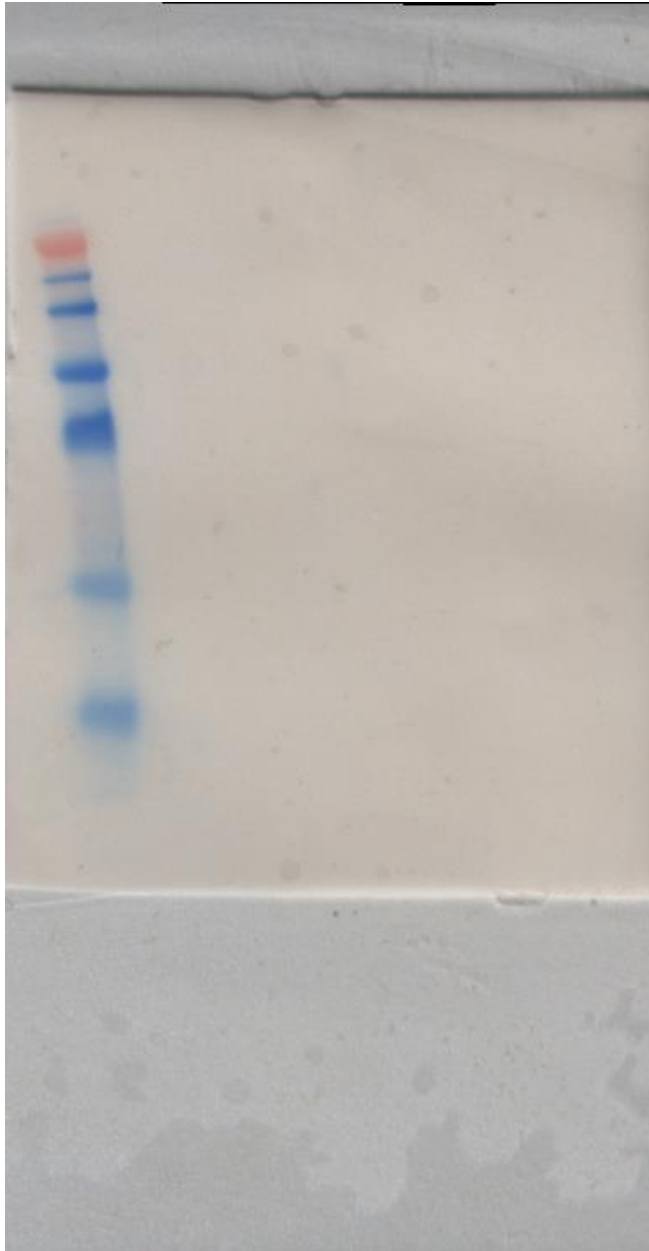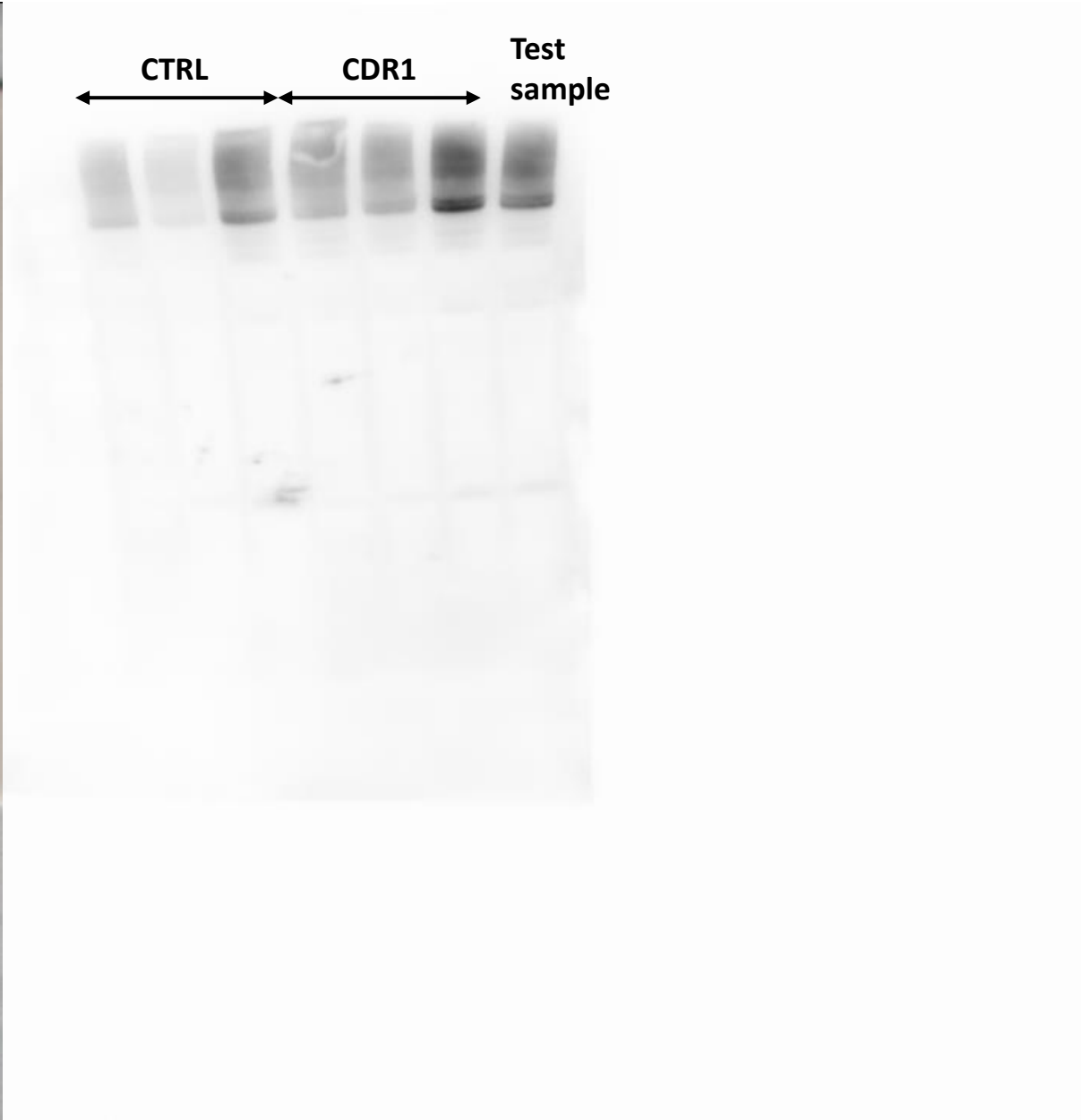

**Histone  
acetylation**

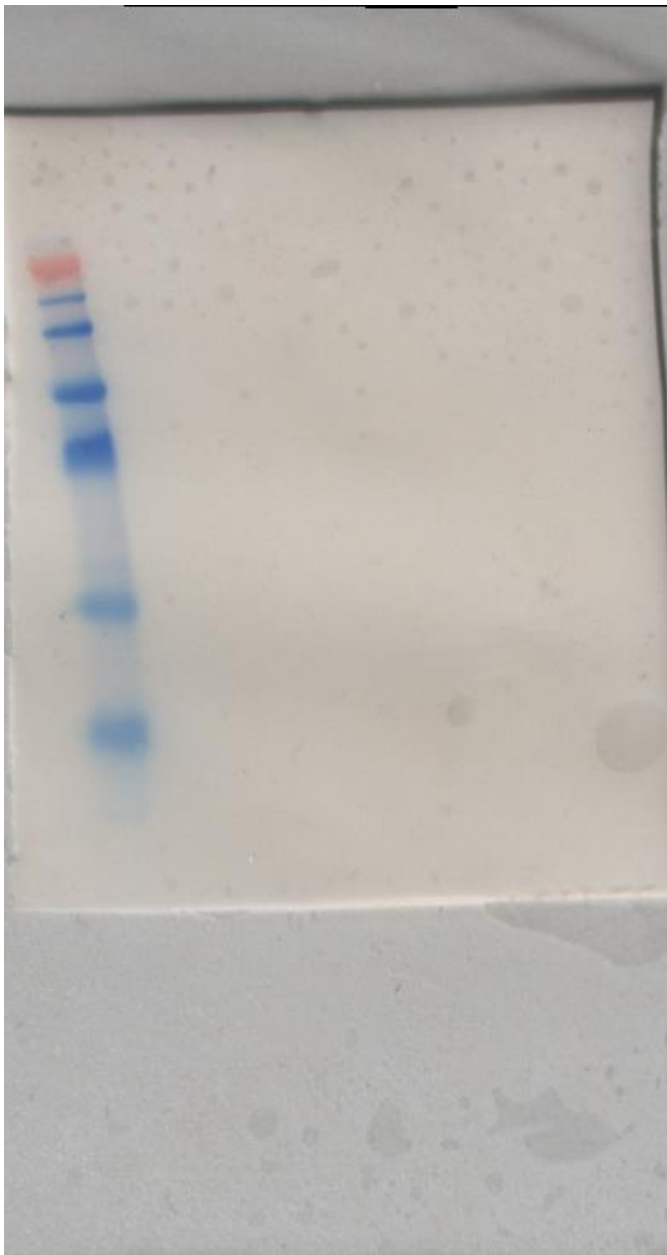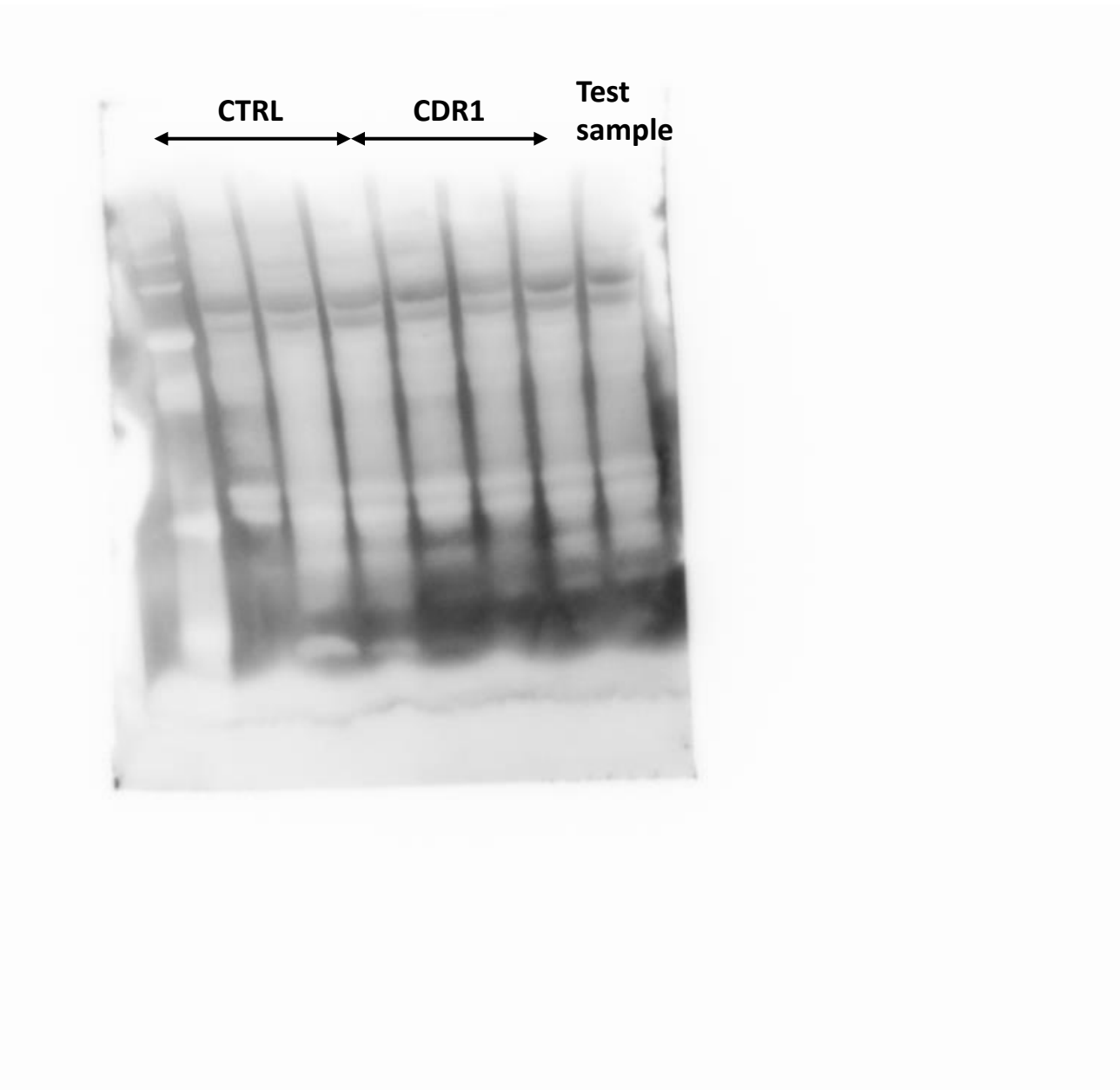

Supplement: Supplementary file 1 [file biomolecules-13-01161-s001.zip › File S1.pdf]

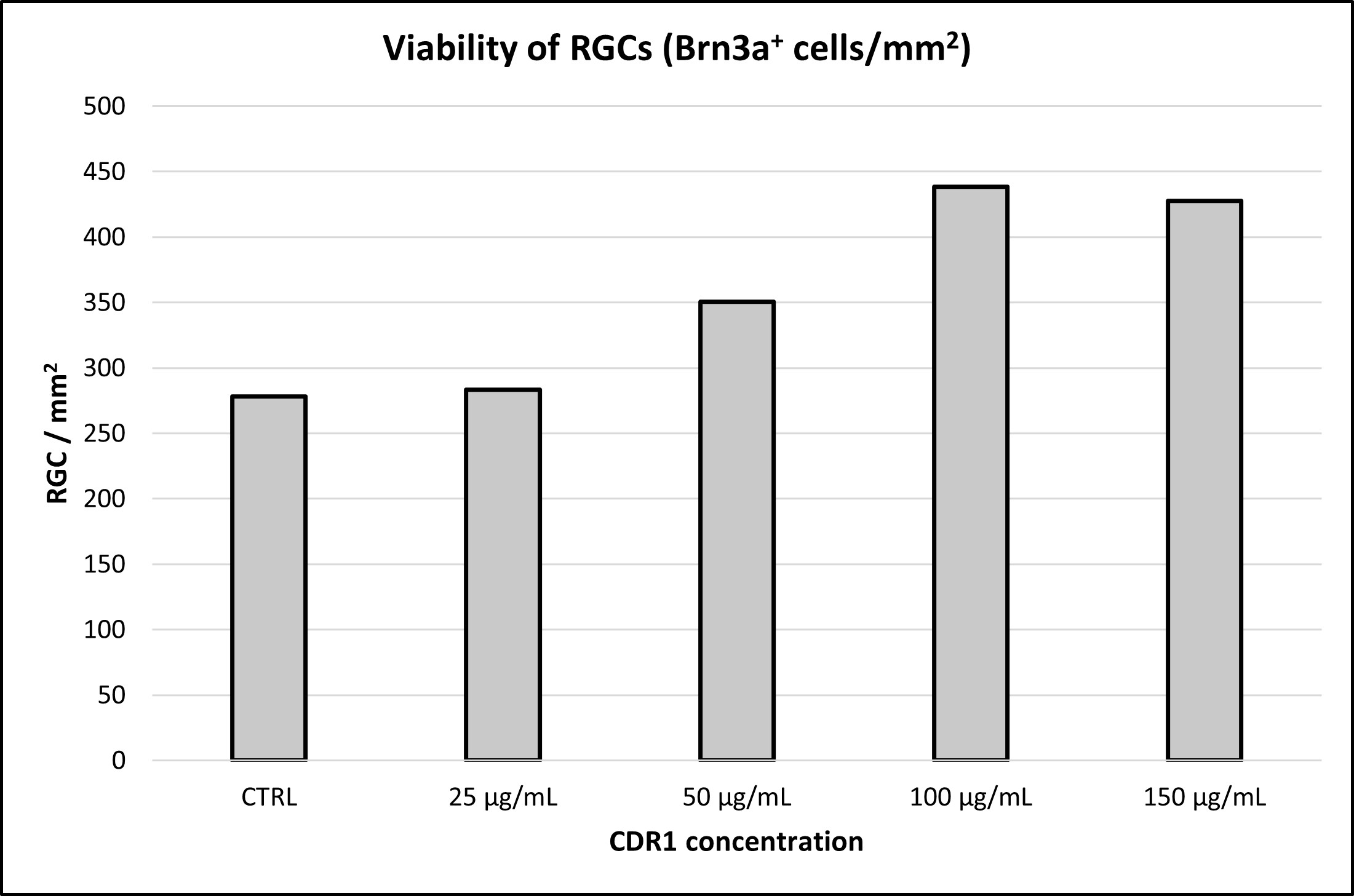

Supplement: Supplementary file 1 [file biomolecules-13-01161-s001.zip › Supplementary Figure S1.tif]
